# Design and ex vivo development of a suprachoroidal spacer implant to treat glaucoma

**DOI:** 10.21203/rs.3.rs-3895533/v1

**Published:** 2024-01-31

**Authors:** Bryce Chiang, Kyeongwoo Jang, Jeffrey Goldberg, David Myung

## Abstract

Glaucoma is a leading cause of visual impairment and blindness in the United States and worldwide. Elevated intraocular pressure (IOP) has been identified as the only modifiable risk factor in glaucoma, and there exists a need for a glaucoma procedure that is safe, efficacious, and can be performed in the outpatient clinic setting. Suprachoroidal expansion has been explored as a method to lower IOP previously. The purpose of this work was to design a monolithic hydrogel implant that would not clear or degrade to potentially achieve long term (possibly permanent) IOP reduction. Here, we developed and showed ex vivo testing of a novel photo-crosslinked polyethylene glycol (PEG) suprachoroidal spacer implant delivered via a custom-designed injector system. We optimized the composition, shape, and mechanics of the implant to be suitable for implantation with the suprachoroidal space. We developed a microneedle injector system to deliver this implant. We showed precise control over implant location and volume occupied within the suprachoroidal space. Further preclinical testing is needed to demonstrate efficacy.

## 1. Introduction

Glaucomatous optic neuropathy is a leading cause of visual impairment and blindness in the United States and worldwide[1]. The optic nerve, comprising 1.2 million retinal ganglion cell axons, transmits all visual information from the eye to the brain. Glaucoma is characterized on exam by progressive cupping of the optic nerve head, perceived by patients as slow, painless, and progressive loss of peripheral vision that can eventually affect central vision.

Elevated intraocular pressure (IOP) has been identified as the only modifiable risk factor in glaucoma, and IOP reduction of 20–30% has been shown to slow or halt the progression of glaucoma[2–7]. IOP is determined by the balance of aqueous humor production and elimination[8], with a population average of 14.5 mmHg classified as 'normal' and > 21 mmHg as 'abnormal' (two standard deviations above normal). Current IOP reduction strategies include topical eye drops, laser procedures, and surgeries. Topical eyedrops, with various mechanisms of actions such as lowering aqueous production and/or increasing outflow, are effective and generally well tolerated. However, medication adherence is low, often related to increasingly complex medication regimens, tolerability of the medicines, dexterity, and/or memory problems[9,10]. Laser procedures can be effective, but the magnitude and duration of IOP reduction are unpredictable[11]. There are risks associated with surgery including bleeding, infection, IOP too high or too low, further vision loss, diplopia, etc[12]. Some of these complications can be devastating and result in total loss of vision and/or the eye. Thus, there exists a need for a glaucoma procedure that is safe, efficacious, and can be performed in the outpatient clinic setting.

Studies have shown that suprachoroidal expansion effectively reduces IOP in rabbits and rhesus macaques[13–15]. The suprachoroidal space (SCS), a potential space between the sclera and the choroid, has become an attractive option for targeted ocular drug delivery[15,16]. Expansion of this space is thought to enhance outflow and/or reduce aqueous humor production[15]. The degree of suprachoroidal expansion is proportional with IOP reduction[14]. Notably, expansion with silicone oil does not lower IOP, suggesting that water permeability of the injected material is important, perhaps to allow aqueous humor to freely traverse through the expanded space[15]. Chiang et al. showed that suprachoroidal expansion with carboxymethyl cellulose lowered IOP for up to 1 wk[13]. In other data, a crosslinked in situ-forming hyaluronic acid hydrogel was injected into the SCS to achieve IOP reduction for up to 4 months[14].

These data suggest that a monolithic hydrogel implant that will not clear or degrade has the potential to achieve long term (possibly permanent) IOP reduction. Here, we developed a novel photo-crosslinked polyethylene glycol (PEG) suprachoroidal spacer implant delivered via a custom-designed injector system. This *ex vivo* study focuses on optimizing the injector and implant parameters to safely deliver the implant into the SCS, to lower IOP without causing tissue damage.

### 2. Materials and methods

## 2.1. Materials

PEG derivatives were purchased from Creative PEGWorks, Chapel Hill, NC. Enucleated fresh rabbit eyes were acquired from Pel-Freez Biologicals, Rogers, AR. Other materials were obtained from Sigma Aldrich unless specified otherwise.

## 2.2. Preparation of suprachoroidal spacer implant based on PEG hydrogels

A 30% (w/v) solution of 4-arm PEG methacrylate with a molecular weight (MW) of 10 kDa was prepared in phosphate-buffered saline. 1% (w/v) lithium phenyl (2,4,6-trimethylbenzoyl) phosphinate (LAP) was added as a photo-initiator. For compression testing, this pre-gel solution was injection molded into 24-well plates. For *ex vivo* delivery, the pre-gel solution was injected into clear silicone tubing (0.3 mm inner diameter (ID)) of various lengths (15, 30, 37.5, and 45 mm). The solutions were then exposed to ultraviolet light (365 nm wavelength) for 5 min to initiate photo-polymerization, forming transparent crosslinked PEG hydrogels.

In some cases, 1% fluorescein was added to the pre-gel solution as a tracer dye to better visualize the implant. For the PEG hydrogels prepared in silicone tubing, a stainless-steel rod (11 – 0, 0.279 mm diameter, MicroGroup, Medway, MA) was used to push the PEG implant out of the mold. In some cases, the tubing was connected to the distal bore of the suprachoroidal spacer implant injector, and the rod was used to load the implant into the injector.

For optimization, various parameters were varied, including the arrangement of methacrylate functional groups in PEG, MW of PEG (5, 10, and 20 kDa), weight percentage (w/v) of PEG (10, 20, 30, and 40%), ID

of silicone tubing (0.3, 0.5, and 1.0 mm), and length of silicone tubing (15, 30, 37.5, and 45 mm). The final manufacturing process is described above.

### 2.3. Mechanical testing of bulk PEG hydrogels and suprachoroidal spacer implant

Blocks of PEG hydrogels were tested in compression on an Instron 5565 Universal Testing Machine using a protocol similar to the ASTM D695 standards (Compression of Rigid Plastics). Briefly, the PEG hydrogels were fabricated into a cylinder with diameter of 16 mm and height of 1–2 mm. The Universal Testing Machine was set to compress the material at a rate of 50 mm/min. Triplicate blocks of materials were tested in a hydrated state.

Due to the small size of the suprachoroidal spacer implant candidates (PEG hydrogels made with 0.3, 0.5, and 1.0 mm diameter), it was not possible to directly test the mechanical properties of the implant. Thus, the bend strength of the implant candidates was determined instead. The implants were held 5, 10, 20, and 30 mm away from the bottom tip, and the tip was applied perpendicularly to a balance scale. The maximum force of the implant was recorded for 5 different segments. The same procedure was performed with the PEG hydrogels in their hydrated and dehydrated forms. In some cases, the PEG hydrogel was dip coated in Viscoat (Alcon, Fort Worth, TX) for 5 min and then dried.

### 2.4. Fabrication of suprachoroidal spacer implant injector

A 2-inch segment of 27-gauge 304 steel tubing (0.305 mm ID and 0.406 mm outer diameter (OD); MicroGroup, Medway, MA) was used to make the custom-designed injector (Fig. 1). A 45° bias bevel was cut by wire electrical discharge machine (MV2400S Wire EDM, Mitsubishi Electric, Chiyodi City, Japan) and polished on 400 grit sandpaper. Eight hundred micrometers (800 μm or 0.8 mm) posterior to the needle tip, a stainless-steel washer (#000, 1.02 mm ID; McMaster-Carr, Santa Fe Springs, CA) was soldered to the tubing with silver flux. Care was taken to avoid soldering the needle tip. A stainless-steel rod (11 – 0, 0.279 mm diameter; MicroGroup, Medway, MA) was used as a plunger to deliver the implant through the bore of the injector.

### 2.5. Optimization of suprachoroidal spacer implant injector

To determine the ideal length and insertion angle of the needle, various injectors were fabricated with different angles of bevel (25° and 45°), stainless steel washer diameter (#00 and #000), and length of microneedle (0.6, 0.8, 1.0, and 1.2 mm). These injectors were applied to the sclera of enucleated fresh rabbit eyes until a ‘pop’ was felt. A metal rod was introduced through the bore of the injector and slowly advanced to determine whether the injector was placed in the SCS or not. Four replicates were performed for each condition. The final manufacturing process is described above.

### 2.6. Determination of force to penetrate choroid and retina: optimization of PEG hydrogel implant parameters

To determine the force needed to penetrate through the choroid/retina, the following was performed to indirectly determine the force needed by proxy. First, fresh enucleated rabbit eyes were prepared, and a 27G microneedle with length of 0.8 mm was applied to the sclera perpendicularly until a 'pop' was felt. Prolene sutures of different sizes (2 – 0, 4 – 0, 5 – 0, 6 – 0, 7 – 0, 8 – 0, 9 – 0, and 10 – 0; Ethicon, Bridgewater, NJ) were introduced through the bore of the microneedle. Whether the suture penetrated choroid/retina or not was recorded. Six to eight replicates were done for each suture diameter. Subsequently, Prolene sutures of different sizes were held 5, 10, 20, or 30 mm away from the bottom tip and applied perpendicularly to a balance scale. The maximum force was recorded for 5 segments.

## 2.7. *Ex vivo* delivery of suprachoroidal spacer implant

Figure 2 illustrates the *ex vivo* delivery process of the PEG implant into the SCS of enucleated fresh rabbit eyes. Initially, the PEG hydrogel implant was polymerized and loaded into the custom-designed injector. The injector was inserted into the sclera until a 'pop' was felt. A metal rod was used to advance the implant through the injector bore into the SCS, after which the injector was removed.

In some cases, ultrasound biomicroscopy (UBM) was used to verify implant delivery. The eyes were then flash frozen in ethanol chilled over dry ice, dissected in a petal fashion as previously described[17], and illuminated with blue light to visualize the fluorescein-dyed implant.

## 2.8 Statistical analysis

Statistical analysis was conducted using Matlab (Mathworks, Natick, Massachusetts), Microsoft Excel (Microsoft Corp., Redmond, Washington), Origin (OriginLab Corporation, Northampton, Massachusetts), and GraphPad Prism (GraphPad Software, San Diego, California). Mean ± standard deviation is presented where appropriate. The significance of probabilities was compared on a binomial distribution against a 50% success rate. Paired Student t-test with Holm-Sidak multiple comparison correction was used to compare means. When appropriate, Chi-squared analysis was used to compare frequencies of occurrences. A statistical significance level of 0.05 was set *a priori*.

### 3. Results and discussion

## 3.1. Design of suprachoroidal spacer implant

The suprachoroidal spacer implant should be: (1) water permeable, facilitating free water flow within the SCS; (2) sufficiently rigid to easily advance within the SCS; (3) not so rigid as to inadvertently penetrate the choroid and retina; (4) capable of remaining within the SCS long term; and (5) most importantly, non-toxic and biocompatible.

To meet these criteria, we designed the implant as a monolithic crosslinked hydrogel based on PEG derivatives (Fig. 3). PEG is recognized as biologically safe by the United States Food and Drug Administration (FDA) and is widely used in various fields including, chemical, cosmetics, food, and pharmaceutical industries[18,19]. In particular, crosslinked PEG-based hydrogels have shown therapeutic potentials due to the biocompatibility, permeability, and negligible cytotoxicity[20,21]. The fabrication of ideal PEG-based hydrogels as a suprachoroidal spacer implant was optimized by testing different hydrogels prepared with different parameters including arrangement of methacrylate functional groups (PEG dimethacrylate and 4-arm PEG methacrylate), MW of PEG (5, 10, and 20 kDa), and weight percentage (w/v) of PEG (10, 20, 30, and 40%) in pre-gel solutions.

## 3.2. Mechanical testing of PEG hydrogels

We tested cylindrical PEG hydrogel blocks, fabricated with varying parameters, for stiffness at 5% strain in compression mode. Hydrogels made from 4-arm PEG methacrylate (10 kDa MW, 30 weight %) showed higher stiffness compared to those made from PEG dimethacrylate (Fig. 4A). This is likely because the hydrogels prepared with 4-arm PEG methacrylate became more resistant to deformation due to increased crosslinking density. Further tests varying the MW and weight % (w/v) of 4-arm PEG methacrylate showed that a lower MW, with a constant weight %, resulted in higher stiffness due to a greater number of crosslinking sites (Fig. 4B). Similarly, a higher weight % (w/v) in pre-gel solutions led to stiffer hydrogels (Fig. 4C).

Because of the small dimensions of the suprachoroidal spacer implant candidates (PEG hydrogels made with 0.3-, 0.5-, and 1.0- mm diameter), direct mechanical testing was not possible. Thus, bend strength of PEG implants with different parameters was determined (Table 1). The parameters that seemed to impart the greatest rigidity to the PEG implants were a diameter of 1.0 mm and relative dehydration of the implant. However, neither of these were feasible, as it was observed that upon contact with water, the PEG implants rapidly rehydrated within seconds and lost the mechanical rigidity of the dehydrated implants. Furthermore, 0.5- and 1.0- mm diameters were larger than the bore of the needle. No PEG implants with diameter of 0.3 mm were able register a force before bending.

Table 1 Bending force of select PEG hydrogels with length of 30, 20, 10, and 5 mm, indicating the material's rigidity. Mean ± SD for triplicates.

| Diameter | Hydrogel conc | Dipcoating | State | Force at 30mm (mg) | Force at 20mm (mg) | Force at 10mm (mg) | Force at 5mm (mg) |
|---|---|---|---|---|---|---|---|
| 1.0mm | 0.5 | Viscoat | Wet | 0 ± 0 | 210 ± 10 | 720 ± 15 | 1930 ± 570 |
| 1.0mm | 0.5 | Viscoat | Dry | 0 ± 0 | 5750 ± 2480 | 11070 ± 450 | 16590 ± 1210 |
| 1.0mm | 0.5 | None | Wet | 230 ± 30 | 330 ± 60 | 910 ± 200 | 3700 ± 520 |
| 1.0mm | 0.5 | None | Dry | 0 ± 0 | 1730 ± 440 | 3490 ± 390 | 6090 ± 2700 |
| 0.5mm | 0.3 | 2% CMC | Wet | 0 ± 0 | 0 ± 0 | 19 ± 1 | 61 ± 15 |
| 0.5mm | 0.3 | 2% CMC | Dry | 0 ± 0 | 0 ± 0 | 33 ± 6 | 43 ± 2 |
| 0.5mm | 0.5 | None | Wet | 11 ± 6 | 11 ± 5 | 33 ± 11 | 55 ± 11 |
| 0.5mm | 0.5 | None | Dry | 0 ± 0 | 26 ± 2 | 44 ± 4 | 71 ± 4 |
| 0.3mm | 0.4 | Viscoat | Wet | 0 ± 0 | 0 ± 0 | 0 ± 0 | 0 ± 0 |
| 0.3mm | 0.4 | Viscoat | Dry | 0 ± 0 | 0 ± 0 | 0 ± 0 | 0 ± 0 |
| 0.3mm | 0.4 | None | Wet | 0 ± 0 | 0 ± 0 | 0 ± 0 | 0 ± 0 |
| 0.3mm | 0.4 | None | Dry | 0 ± 0 | 0 ± 0 | 0 ± 0 | 0 ± 0 |

### 3.3. Development of custom-designed injector

The PEG-based implant was delivered into the SCS with our custom-designed injector (Fig. 1). The injector enables the implant to safely and reliably access the SCS using a microneedle with length matched to the thickness of the sclera[22,23]. A 27-gauge needle (0.406 mm OD, 0.305mm ID) was chosen as this is the largest commonly used needle size for intravitreal injections in the outpatient ophthalmology clinic[24]. Such a needle size is commonly used and does not require suturing of the sclerotomy to prevent leakage and/or infection. Prior studies using a microneedle to deliver drugs into the SCS were used perpendicular to the sclera, but such an approach would likely kink the implant and/or penetrate the choroid/retina. Thus, an oblique approach was used.

To determine the ideal length and insertion angle of the needle[16,22,23], we performed insertion studies in *ex vivo* rabbit eyes (Fig. 5). Injectors with different needle lengths were applied to sclera and a metal rod was advanced through the bore of the injector. If strong resistance was felt and the injector came off the eye, the tip of the injector had not yet entered the eye (outside the eye, indicated by a yellow V). If the rod could be easily advanced and a slight ‘pop’ was felt (rod going through choroid/retina), the tip of the injector was considered to be in the SCS (indicated by a green O). If the rod could be easily advanced and no ‘pop’ was felt and the rod was visible within the eye, the tip of the injector was considered to be in the vitreous (indicated by a red X). It was observed that a needle length of about 1.0 mm at an oblique angle was able to reliably enter the SCS. Such an oblique angle would enable delivery of the spacer implant without kinking. From this experiment, we derived the optimized parameters for the suprachoroidal injector, i.e., 27G bore, microneedle length of 0.8 mm, 45° needle bevel, and 34° angled inserter (Fig. 1B).

### 3.4. Optimization of PEG hydrogel implant parameters for atraumatic delivery to the SCS.

To improve the reliability of the implant delivery into the SCS without damaging the choroid or retina, the force required to penetrate the choroid/retina was determined using fresh enucleated rabbit eyes. As a proxy, different sized Prolene sutures (2 – 0, 4 – 0, 5 – 0, 6 – 0, 7 – 0, 8 – 0, 9 – 0, and 10 – 0) were advanced through the bore of the microneedle inserted onto the sclera. If a slight ‘pop’ was felt (suture going through choroid/retina), this Prolene suture was recorded as being able to penetrate through the choroid/retina. If no ‘pop’ was felt with a Prolene suture, it was recorded as unable to penetrate through the choroid/retina. If the suture could not be advanced, another sclerotomy was made and this was not considered a replicate.

Figure 6 shows the probability of these Prolene sutures penetrating choroid/retina as well as the bend force of each suture. Prolene sutures designated 6 – 0 (diameter 90 µm) were unlikely to penetrate choroid/retina, and 7 – 0 (diameter 67 µm) never penetrated in our study. The 7 – 0 Prolene suture had a bend force of 0.262 ± 0.035 gF when held 5 mm from the tip. This force was considered as the upper limit and used to guide the choice of the optimized PEG hydrogel implant parameters. Since the 0.3 mm diameter PEG hydrogels were not able to register a bend force, the final parameters were chosen as follows: 30% (w/v) 4-arm PEG methacrylate, 10 kDa MW, and 0.3 mm diameter.

## 3.5. *Ex vivo* delivery of suprachoroidal spacer implant

The ability of the custom-designed injector to deliver the spacer implant into the SCS was determined by noninvasive UBM, which has been used previously to examine suprachoroidal expansion[25,26], and dissection[17,27]. After delivery, the UBM image showed the injected implant as a void within the SCS (Fig. 7A-E). The optimized injector was able to reliably deliver the PEG hydrogel implants of 4 different lengths (15, 30, 37.5, and 45 mm). Out of 38 loaded implants, 34 (89.5%) were delivered within the SCS and the other 4 into the sub-retina. Injectors made with other parameters were not as reliable as the optimized design.

Assuming the shape of the injected PEG implant as an ellipsoid, the volume that the PEG implant occupied in the SCS was estimated. First, the thickness of the implant within the SCS was determined from the UBM images. As examples, one UBM image is shown for each different implant length (Fig. 7B-E). Then, the eye was dissected and illuminated by blue light to better visualize the fluorescein dye added to the implants (Fig. 7F and G). The photos of the dissected eyes were analyzed using the Image J software to precisely estimate the major and minor axes of the ellipse occupied by the implant within the SCS. Finally, the total volume of the SCS implant was determined by calculating the volume of an ellipsoid:

$V = \frac{4}{3}\pi abc$, where $a$ = SCS thickness/2, $b$ = radius of major axis, and $c$ = radius of minor axis

The results showed that the thickness, cross-section area, and the total volume of the SCS occupied by the injected implants were proportional to the implant's lengths (Fig. 8). As the implant length increased from 15 to 45 mm, the SCS thickness increased from 0.276 to 0.525 mm, and the estimated volume of the SCS implant increased from 0.403 to 1.76 $mm^3$ (= μL). Interestingly, the experimentally estimated volume of the implant within the SCS was only 44% of the theoretical volume of the cylindrical PEG implant (averaged from 4 different lengths). It is possible that the implant was partially dehydrated or compressed within the tissues post-injection, or the experimentally determined volumes might have been underestimated because the actual geometry of the implant within the SCS may not be precisely ellipsoidal. Despite this discrepancy, this shows we are able to fine tune the extent of SCS expansion, which is likely to influence the degree of IOP reduction, by varying the implant length. This aspect will be further tested in *in vivo* studies.

## 3.6. Clinical insights into suprachoroidal spacer implantation

Previous studies by Chiang et al[13] and Chae et al[14] demonstrated that suprachoroidal expansion with a fluid formulation can lower IOP. However, controlling the extent and direction of suprachoroidal expansion with a liquid formulation is challenging. Our study shows that varying the implant's length can achieve different heights and volumes within the SCS. A solid monolithic implant, like ours, may offer easier implantation that has the advantage of remaining in place and also be removal if desired. Additionally, a recent phase II open label clinical trial demonstrated 39% IOP reduction with an implant inserted into the SCS[28]. The Ciliosleral Interposition Device (Ciliatech, Chavanod, France) is an acrylic-based implant with dimensions (6 mm circumferential, 4 mm anterior-posterior, and 0.2 mm thick plate) similar to our 45mm PEG implant in volume. While the Ciloscleral device requires implantation in the operating room, the hydrogel implant described herein has the potential to be delivered in-office by microneedle injection[29].

It is important to note that the suprachoroidal spacer implant described (as well as the Ciliatech and liquid formulation described previously) are not like other "suprachoroidal"/ "supraciliary" minimally invasive glaucoma surgical devices (MIGS), such as the Cypass®[30,31], MINIject[32,33], among others[34,35]. With those devices, the implant is used to create, and stent open a cyclodialysis cleft. This creates a direct communication between the anterior chamber and suprachoroidal space, enabling shunting of fluid without a subconjunctival bleb. Though effective in lowering IOP[31], there was loss of corneal endothelium that could eventually require corneal transplantation[36]. Ultimately, the Cypass® device was voluntarily withdrawn from the market[37], and other devices are in various stages of development to fill the void[38]. Since the suprachoroidal spacer implant and the suprachoroidal MIGS function by different mechanisms of action, it is possible that they may lower IOP synergistically.

## 4. Conclusions

In this study, we have described the *ex vivo* development of a monolithic hydrogel suprachoroidal spacer implant designed to lower IOP. The SCS, a potential space between the sclera and the choroid held together by fibrils[25], can be expanded to reduce IOP[13–15]. We selected crosslinked PEG hydrogels as the implant material for its ease of manufacturing, mechanical properties, biocompatibility, and permeability. The stiffness of the implant was optimized to allow easy advancement within the SCS without penetrating the choroid/retina.

We also developed a custom-designed injector system for safe implant delivery into the SCS. The 27-gauge microneedle, the largest size used for intravitreal injections in the outpatient ophthalmology clinic, was chosen to eliminate the need for additional sutures post-injection[24]. Our experiments showed that a microneedle length of 0.8 mm was able to reliably access the suprachoroidal space. Microneedles, FDA-approved for drug delivery in the SCS, are typically held perpendicular to the sclera[22,23,39], which require that the injected fluid formulation be able to navigate this angle[22,23]. However, given our implant's need to be delivered without kinking, an oblique needle angle and an overall needle design were necessary. To our knowledge, this is the first use of a microneedle to deliver a solid implant (and not a liquid formulation) into the SCS. Further optimization on the implant molecular makeup and biomechanical properties were done.

In summary, this study describes the development of a suprachoroidal spacer implant and injector system designed to lower IOP and treat glaucoma. The injector and PEG hydrogels' physical properties were optimized for atraumatic delivery of thin cylinders into the SCS, without surgical procedures. The implant tended to coil, likely due to the mechanical properties of the PEG hydrogel. Further design improvements may yield a non-coiling implant. Future research will focus on evaluating the implant's therapeutic potential in preclinical animal studies and eventually in human clinical trials.

### Declarations

## Competing Interests

B.C., J.L.G, and D.M. are inventors on a patent application filed through Stanford University. An approved plan for managing any potential conflicts arising from this arrangement is in place. No potential competing interest was reported by the other authors.

#### Competing Interests

B.C., J.L.G, and D.M. are inventors on a patent application filed through Stanford University. An approved plan for managing any potential conflicts arising from this arrangement is in place. No potential competing interest was reported by the other authors.

## Author Contribution

All authors contributed to the study conception and design. Material preparation, data collection and analysis were performed by B.C and K.J. The first draft of the manuscript was written by B.C. and all authors commented on previous versions of the manuscript. All authors read and approved the final manuscript.

## Acknowledgments

We thank Peter Franco (Stanford National Accelerator Laboratory) for helping with wire EDM machining. This work was supported by the National Eye Institute under grants K08-EY033407 and P30-EY026877, Research to Prevent Blindness, and Stanford Department of Ophthalmology.

## Figures

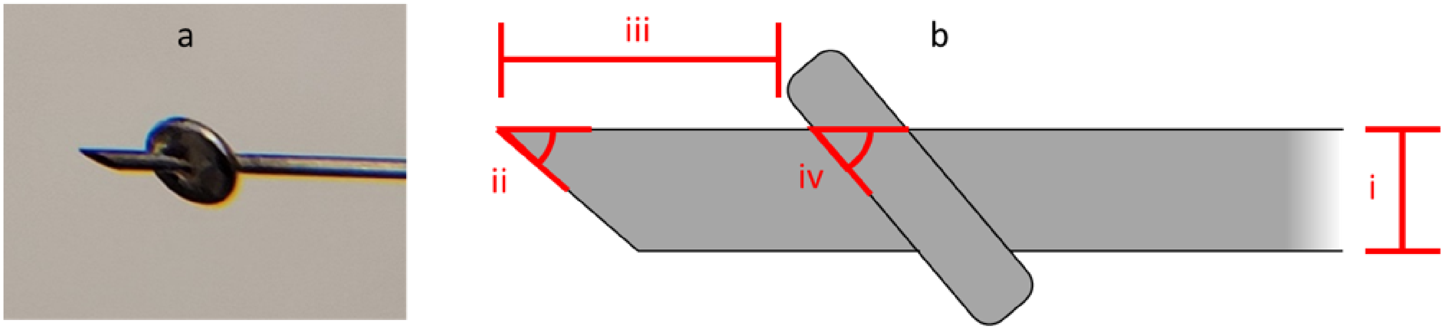

### Figure 1

(A) Photo of custom-designed injector with optimal parameters. (B) Diagram of injector with key parameters labeled: (i) injector gauge, (ii) injector bevel angle, (iii) microneedle length, and (iv) angle of inserter *NOT TO SCALE.

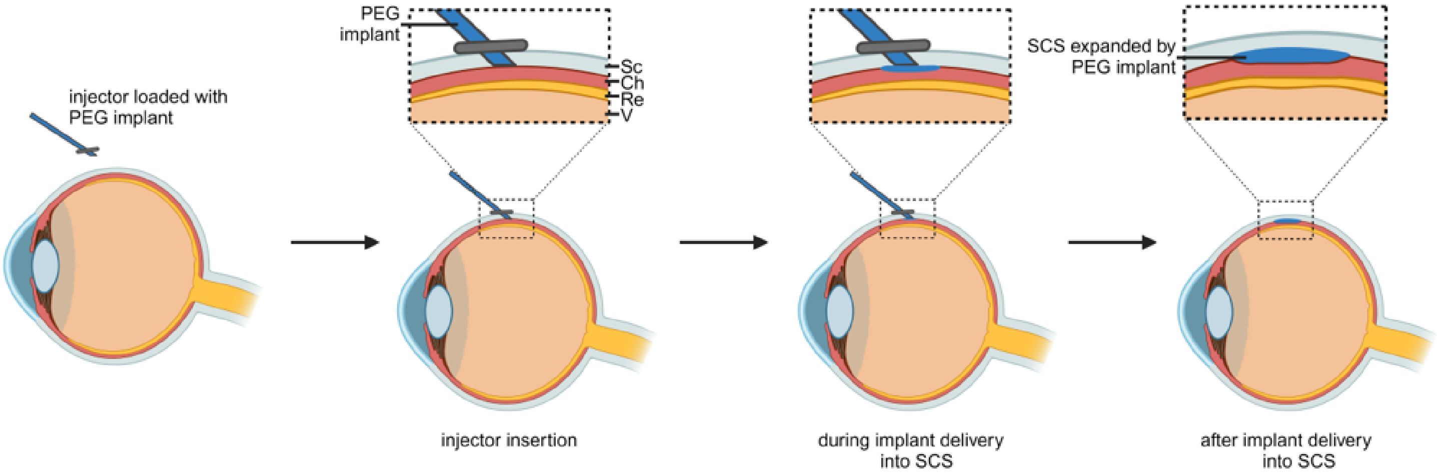

### Figure 2

Illustration demonstrating the delivery of the PEG implant into the SCS of enucleated fresh rabbit eyes. Sc-sclera, Ch-choroid, Re-retina, V-vitreous.

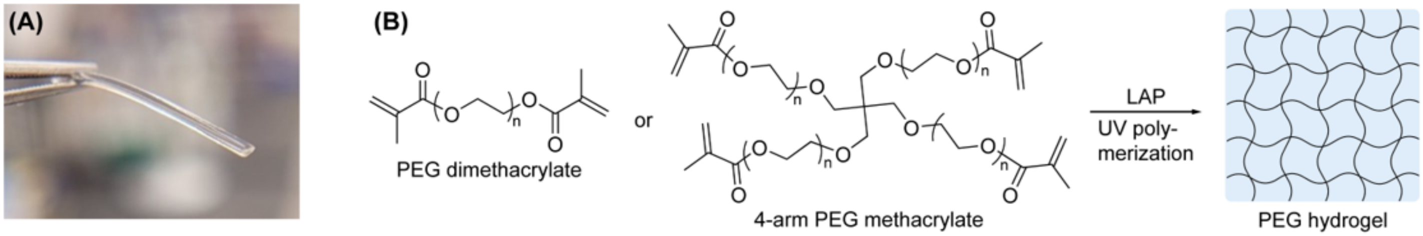

### Figure 3

(A) Photo of PEG hydrogel implant (1.0 mm diameter). (B) Preparation of PEG hydrogels by UV-crosslinking PEG derivatives. The structure of PEG hydrogel shown is a simplified representation of its permeable network.

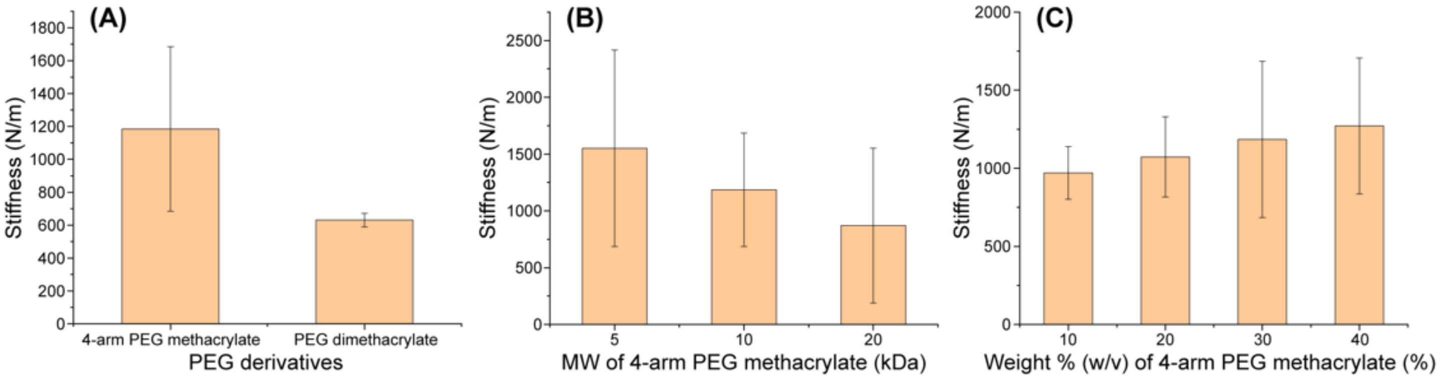

### Figure 4

Graphs of stiffness (Young's modulus at 5% strain) for PEG hydrogels prepared with varying (A) functional group at 10 kDa MW and 30% (w/v), (B) MW at 30% (w/v), and (C) weight % at 10 kDa MW. Error bars represent standard deviations (N = 3).

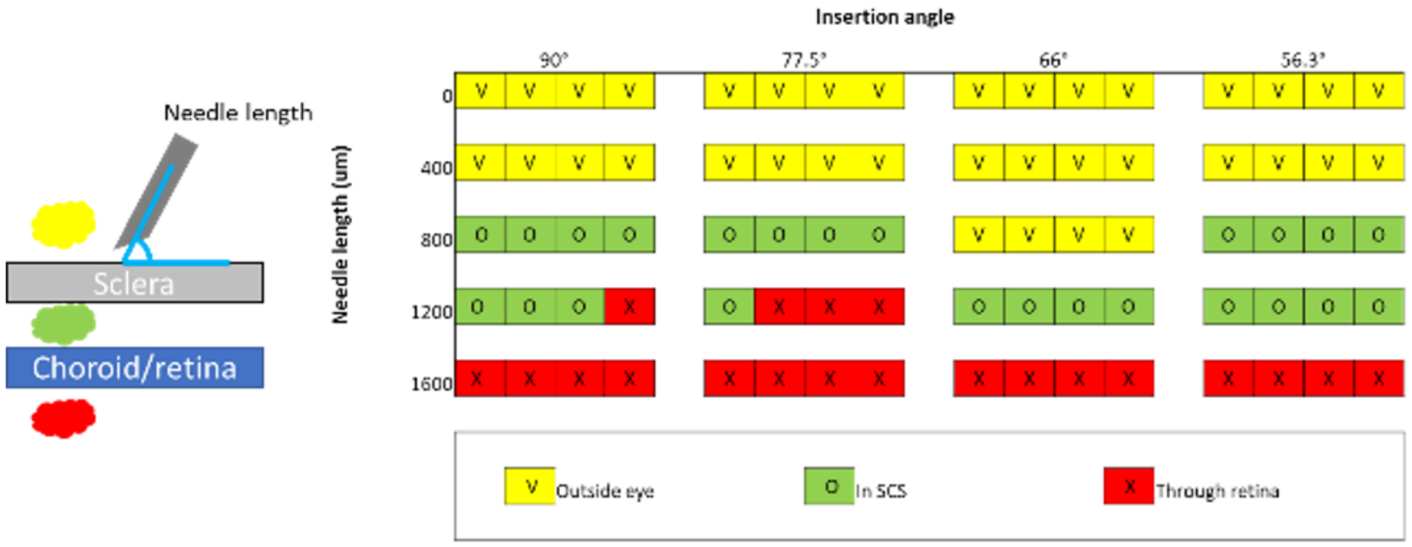

### Figure 5

Illustration and results demonstrating the effect of needle length and insertion angle on implant positioning within the SCS. The tip's location is indicated by different symbols (yellow V, green O, and red X) based on its position. Each square represents one replicate.

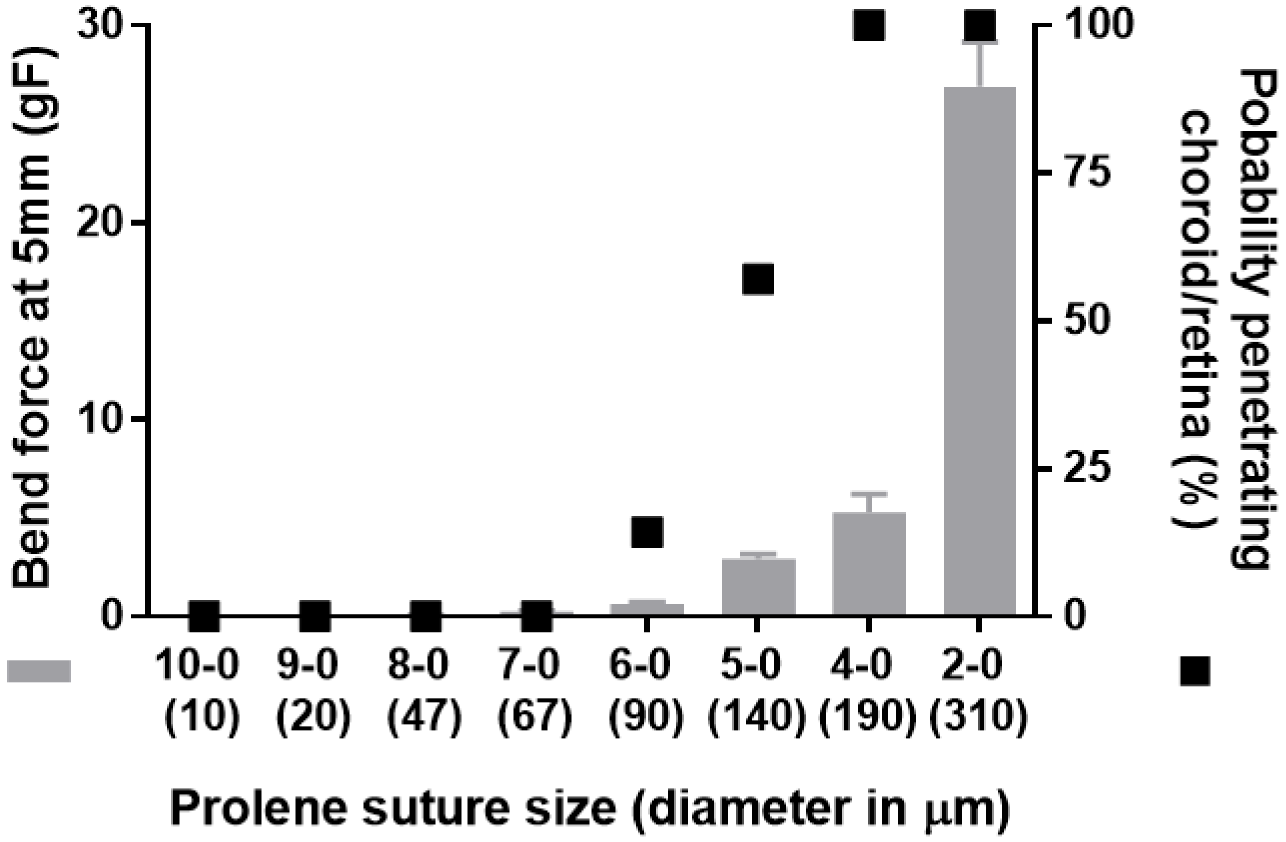

Figure 6

Bend force when suture held at 5 mm (left y-axis with bar graph) and probability of suture penetrating choroid/retina (right y-axis with black square) for different Prolene sutures.

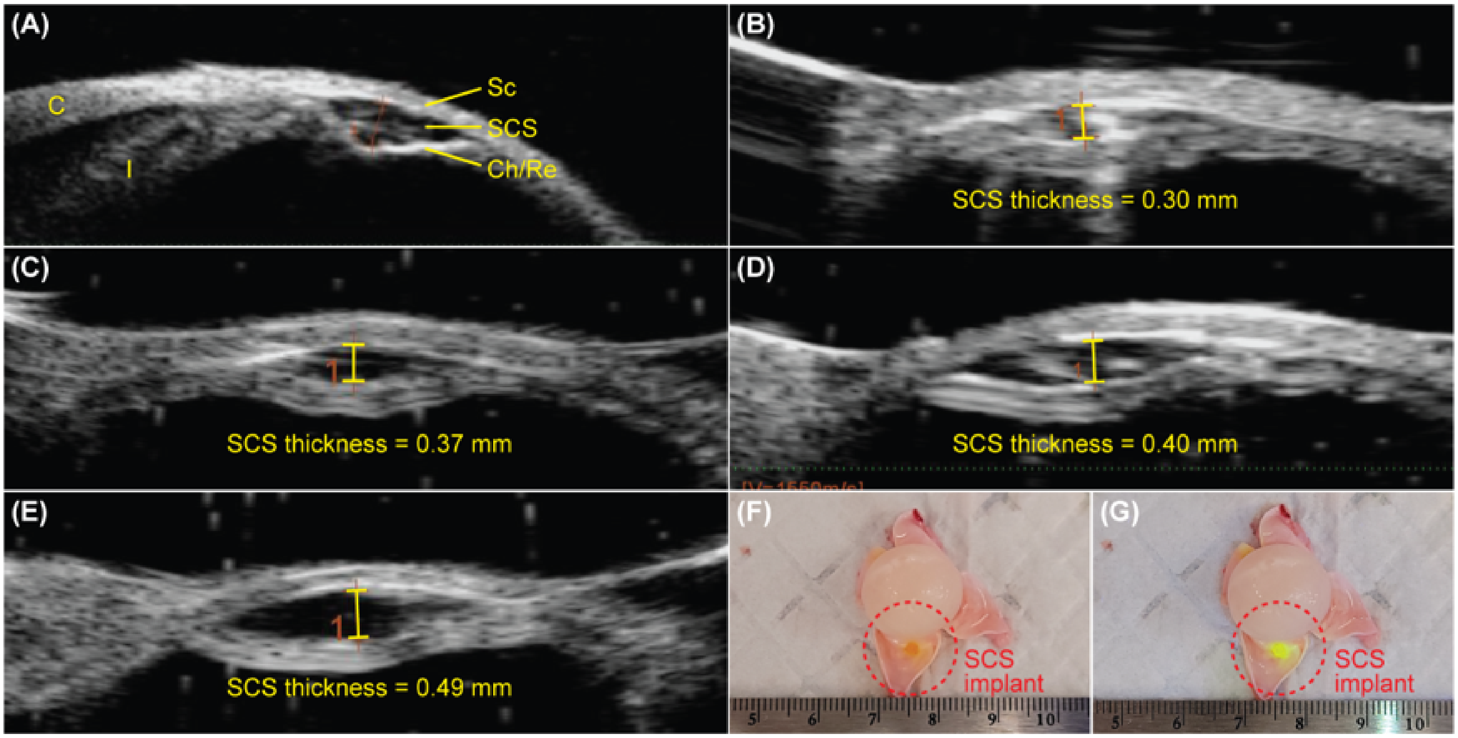

### Figure 7

(A-E) Example UBM images of ex vivo rabbit eye after deploying SCS implant with lengths of 15 mm (B), 30 mm (C), 37.5 mm (D), and 45 mm (E). The SCS thickness was calculated as the maximum perpendicular length of the void between sclera and choroid/retina. (F-G) Example photos of dissected rabbit eye after deploying SCS implant. Same eye (F) without or (G) with blue light. As a visual aid, fluorescein dye was added to the implant. C-cornea, I-iris, Sc-sclera, SCS-suprachoroidal space, Ch/Re-choroid/retina.

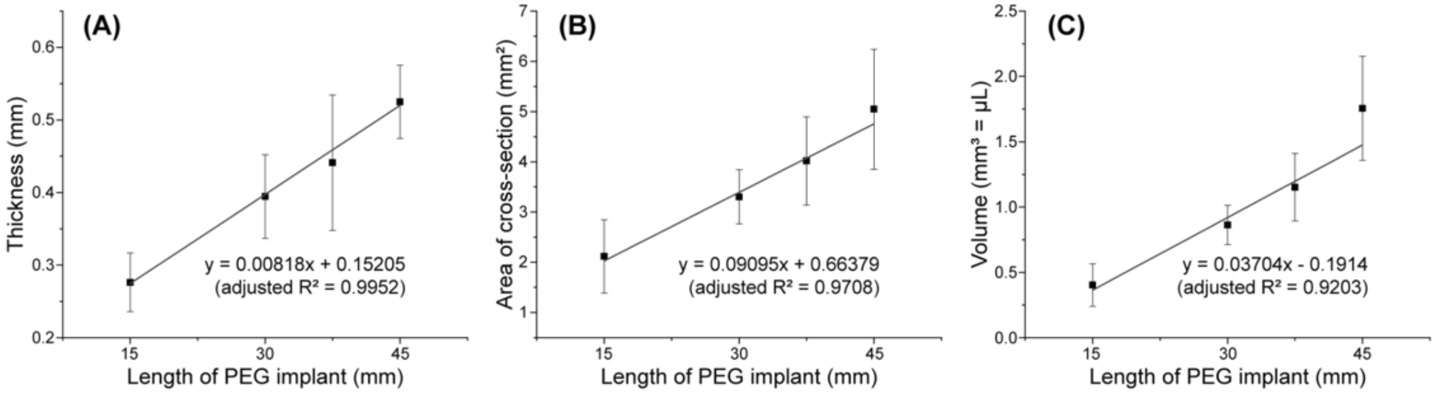

### Figure 8

(A) SCS thickness of implant on UBM, (B) area of cross-section of implant calculated from dissection, and (C) estimated volume of implant within SCS. Error bars represent standard deviations (N = 8 to 9). Red line shows a linear fit.

## Supplementary Files

This is a list of supplementary files associated with this preprint. Click to download.

- Graphicalabstract.png
